# Regional Projections of the Impacts of Future Urbanization and Climate Change on Biogeochemical Cycles in New England Landscapes

**DOI:** 10.34133/research.1043

**Published:** 2025-12-16

**Authors:** Linghui Meng, Afshin Pourmokhtarian, Pamela H. Templer, Lucy R. Hutyra, Jonathan R. Thompson, Charles T. Driscoll

**Affiliations:** ^1^ Department of Civil and Environmental Engineering, Syracuse University, Syracuse, NY 13244, USA.; ^2^ School of Management, Wentworth Institute of Technology, Boston, MA 02115, USA.; ^3^ Department of Biology, Boston University, Boston, MA 02215, USA.; ^4^ Department of Earth & Environment, Boston University, Boston, MA 02215, USA.; ^5^ Harvard Forest, Harvard University, Cambridge, MA 01366, USA.

## Abstract

Human activities have had complex, long-term impacts on forest function across New England—a trend expected to continue. To assess these impacts, we conducted a regional-scale modeling study using the PnET-CN-daily model, simulating multiple scenarios that reflect projected changes in land cover, climate, and air quality. The results suggest that while New England will continue to serve as a regional carbon sink, carbon accumulation in the southern portion of the region will slow and may shift to a net carbon source due to aggressive urban expansion. Carbon dioxide (CO_2_) fertilization and carbon loss associated with urbanization are the dominant factors controlling future carbon dynamics. However, CO_2_ fertilization may diminish over time due to nutrient limitations, while rising temperatures are expected to accelerate soil decomposition, further increasing carbon loss. The forecasts also show that urbanization will increasingly affect ecosystem nitrogen storage. Climate change and CO_2_ fertilization along with declining nitrogen deposition from decreases in fossil fuel use are projected to drive nitrogen oligotrophication—slowing forest growth and becoming more severe as nitrogen inputs decrease. In addition, urbanization and climate change are expected to substantially reduce snowpack and shorten snow cover duration in southern New England, with potential consequences for regional water dynamics. These trends highlight the need to integrate future climate, air quality, and land-use projections into forest management strategies for both urban and rural ecosystems.

## Introduction

The northeastern US forest is an important biome in North America as it plays a critical role in the biogeochemical cycles of water, carbon (C), and nutrients for the region. However, the structure and function of northeastern forests have been substantially impacted by human activities for centuries. Land-use changes such as deforestation, agriculture, and urban expansion have directly reduced the forest extent. Satellite images reveal that New England lost ~129,000 ha (2.8%) of forest cover between 1990 and 2005 [1].

Urbanization also aggravates air pollution and atmospheric deposition. Industrial activities, electricity generation, and vehicle emissions increase the emissions of air pollutants, which coupled with urban heat island effects have exacerbated air quality problems in urban areas. Higher CO_2_ concentrations in urban landscapes compared with those in surrounding rural areas, a phenomenon known as the “urban CO_2_ dome”, has been reported globally [2]. Nitrogen (N) deposition is likewise elevated in urban regions due to concentrated anthropogenic emissions [2]. In contrast, O_3_ exhibits a more complex spatial pattern. Higher emissions of volatile organic compounds associated with human activities and higher air temperature are likely to increase O_3_ formation, while high emissions of nitric oxide (NO) can consume O_3_ and decrease concentrations, resulting in a shorter O_3_ lifetime in urban areas [2]. Consequently, the spatial distribution of O_3_ along an urbanization gradient is largely determined by the NO/NO*_x_* emission rate. In areas with a high NO/NO*_x_* ratio, O_3_ concentrations tend to decrease with increasing impervious surface area (ISA) [3,4].

Urban environments, characterized by higher air temperatures and elevated concentrations of air pollutants, have complex effects on C, N, and water dynamics in ecosystems. Increasing air temperatures [5], elevated atmospheric CO_2_ concentrations [6], and enhanced N deposition [7] can stimulate plant growth. Elevated O_3_ concentrations damage pigments and photosynthetic enzymes, resulting in reduced forest productivity [8]. Enhanced plant growth increases plant N demand, while higher temperatures accelerate soil decomposition, jointly decreasing soil N availability [9]. Elevated N deposition in urban areas can partially offset those soil N losses [10].

Furthermore, water also regulates N cycling by serving as a medium for plant uptake and influencing soil decomposition rates. Higher temperatures increase the vapor pressure deficit and reduce winter snowpack, which can intensify soil water stress [11] and potentially cause root damage during freeze–thaw events [12,13], ultimately altering ecosystem C and N dynamics [11]. In contrast, elevated CO_2_ concentrations reduce stomatal conductance [14], leading to an increase in water-use efficiency (WUE) and a decrease in transpiration [6,14], which mitigates drought stress.

The influence of human activities on forest ecosystems in the northeastern USA is expected to intensify with continued urban expansion and climate change. According to US Census Bureau data, the population in New England grew by 1.2 million (8.6%) between 2000 and 2020, with most of this increase concentrated in Massachusetts (MA) (57.1%), followed by Connecticut (CT) (16.8%), and New Hampshire (NH) (11.9%). The population is projected to continue increasing in the coming decades. Model projections suggest that the percentage of urban land in New England will increase from 10.5% to 18% by 2050 [1].

Long Term Ecological Research in the eastern United States has documented an average annual temperature increase by 0.15 to 0.2 °C per decade, along with an annual precipitation increase by 18 to 29 mm from 1950 to 2019 [15], with a more rapid change observed in recent years [15]. Various modeling studies project a warmer and wetter future climate in New England, although the magnitude of projected increases in temperature and precipitation varies among studies [16].

In this study, we present a modeling framework to characterize and quantify how forests in the northeastern USA may respond to ongoing environmental changes by the mid-21st century, with a particular focus on the complex interactions that occur in urbanized areas. We considered a series of changes in land cover, including an aggressive urbanization scenario driven by substantial population influx but limited urban planning (Growing Global [GG]), a scenario with a similar high population influx but improved urban planning to minimize impacts on surrounding natural areas (Yankee Cosmopolitan [YC]), a scenario in which population and urban expansion continue at the current rate (Recent Trends [RT]), and a reference scenario in which urbanization remains at current levels (Constant scenario). Details of scenario construction are provided in the methods section. Building on our previous work, which quantified changes in air pollutant concentrations and air temperature along an urbanization gradient [2] and the future land-cover scenarios described above for New England [1], we also developed projections of future urban climate and air quality scenarios.

To project ecosystem responses to these future scenarios, we applied the new PnET-CN-daily model, an updated version of the well-established PnET-CN model that has been widely used for ecosystem simulations in the region for decades. The PnET-CN model requires relatively simple input data while simulating complex ecosystem processes, making it well suited for regional-scale applications. In PnET-CN-daily, the simulation time step was refined from monthly to daily to improve process resolution and model accuracy [17]. Using this enhanced model, we projected changes in C, N, and water across New England under a range of future scenarios and additionally quantified the distinct contributions of climate, land cover, and atmospheric chemistry to these ecosystem dynamics.

## Results

### Model calibration

Model calibration was conducted for both hardwood and boreal forests. Model calibration and testing for the hardwood forest (Harvard Forest) site is summarized in an earlier paper [17]. Results from the boreal forest calibration, based on comparisons between model simulations and observations at the Howland Forest conifer stands, suggest that the PnET-CN-daily model effectively simulates the annual patterns of gross primary production (GPP), respiration, and wood mass (Table S1). The model closely matched observed values and temporal variations, with normalized mean absolute error (NMAE) values of 0.09 for GPP, 0.12 for respiration, and 0.05 for aboveground biomass (AGB). Although the annual variation in net ecosystem production was not captured perfectly (NMAE 0.21), the predicted values were close to the observations. Time-series observations of root biomass, soil organic matter (SOM), and soil organic nitrogen (SON) dynamics are not available at Howland. However, the difference between average observations and model simulations is less than 10% for these values.

The regional average simulated AGB is 10,232 ± 1,634 g C·m^−2^, compared to an observed average of 9,255 ± 5,149 g C·m^−2^. The normalized absolute error is 0.11, and NMAE is 0.43, indicating that the model slightly overestimates AGB at the regional scale with moderate deviation. The spatial patterns of percent bias (PBIAS) reveal that overestimation is particularly evident in southern Maine (ME) and southeastern MA (Fig. S1). When stratified by elevation, %ISA, and deciduous fraction, AGB bias increases with elevation (Table S2). While observations indicate an upward trend in AGB with increasing elevation, the model simulated a decline, indicating that it overestimates the negative effects of elevation on forest growth, possibly due to high-elevation forests recovering from acid deposition [18].

The regional average simulated SOM is 16,682 ± 3,569 g C·m^−2^, compared to an observed average of 14,178 ± 6,110 g C·m^−2^. The normalized absolute error is 0.17, and NMAE is 0.40, indicating that the model overestimates SOM at the regional scale, with a moderate level of deviation. The spatial patterns of PBIAS show that overestimation is particularly evident in northern regions, while underestimation occurs primarily in southern urban areas (Fig. S2). Stratification by elevation, %ISA, and deciduous fraction indicates that SOM bias increases with increasing ISA (Table S3) and decreasing deciduous fraction (Table S4).

### Future changes in C storage in New England under land-cover and climate change scenarios

**Fig. 1.**
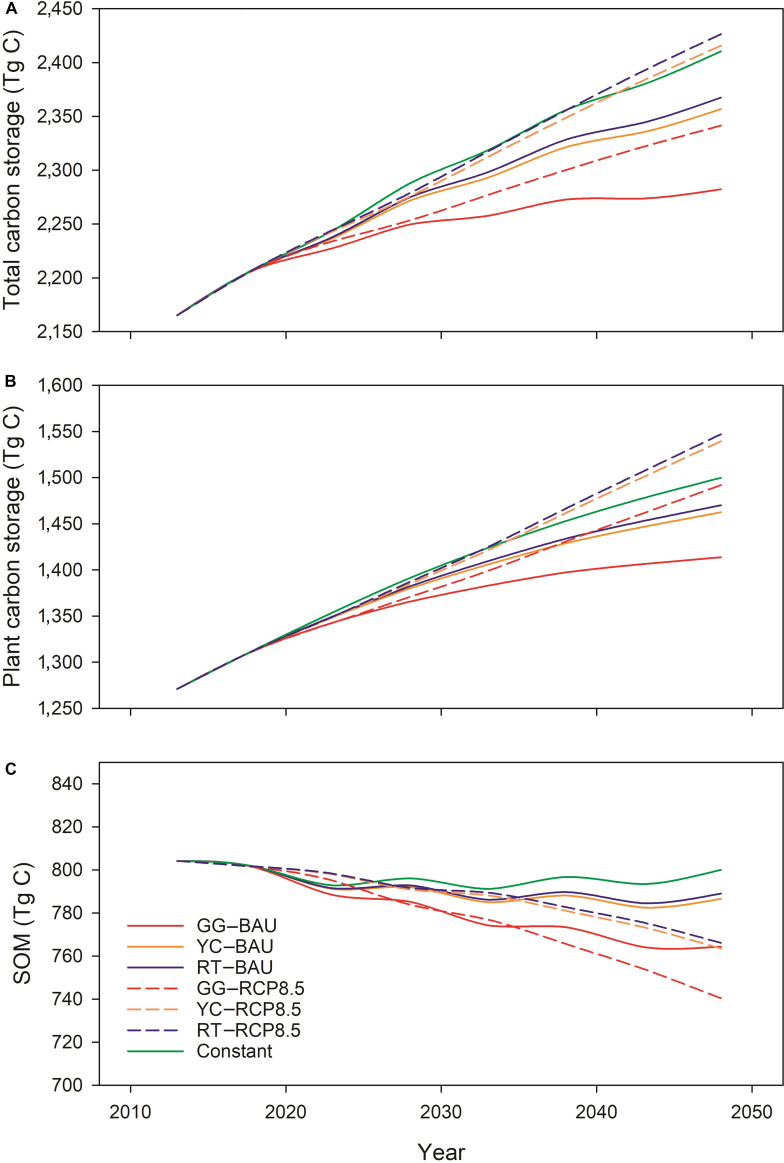
Time series of future projections of total ecosystem net carbon storage (A), plant carbon storage (B), and soil organic matter storage (C) for New England under different land-cover and climate scenarios from 2020 to 2050. GG, Growing Global scenario; YC, Yankee Cosmopolitan scenario; Constant, constant growth scenario; BAU, Business as Usual; RCP8.5, Representative Concentration Pathway 8.5; SOM, soil organic matter.

Projections of the spatial distribution of total ecosystem C storage exhibit considerable heterogeneity across New England. In 2020, the majority of C was stored in ME (51.2%), followed by NH (14.9%), Vermont (VT; 14.8%), MA (11.0%), CT (6.4%), and Rhode Island (RI; 1.8%). It is notable that RI has the highest C density (15.7 kg C·m^−2^), followed by NH (13.5 kg C·m^−2^), ME (13.0 kg C·m^−2^), VT (12.6 kg C·m^−2^), MA (12.4 kg C·m^−2^), and CT (11.8 kg C·m^−2^). The relatively high value for RI may reflect an overestimation of AGB in coastal forest regions (see the “Future changes in carbon storage in regional simulations in New England” section). Model projections indicate a continuous accumulation of the total ecosystem C in New England forests through 2050. Although SOM shows a slight decline, this decline is offset by the increase in plant C storage (Fig. 1). The total ecosystem C storage in New England forests across all future scenarios (except the Constant scenario) is projected to increase from 12.9 ± 0.1 to 14.2 ± 0.1 kg·m^−2^ by 2050, reflecting an average 9.6% increase over the model simulation period (2020 to 2050). The plant and total C trajectories closely mirror each other. Plant C storage across all future scenarios is projected to increase from 7.9 ± 0.1 kg·m^−2^ in 2020 to 9.3 ± 0.3 kg·m^−2^ in 2050, an average of 17.6% increase. Conversely, SOM shows a declining trend under all scenarios, decreasing from 5.0 ± 0.1 to 4.8 ± 0.1 kg·m^−2^ by 2050, an average 4% decrease. We find that the different air quality scenarios (C1 and Clean Energy Futures [CES40B]; see Methods) have a minimal impact on regional C pools. Therefore, these 2 scenarios are combined for the remaining analyses we consider here.

Annual patterns indicate that under a given air quality and land-cover scenario, total ecosystem C storage is greater under the Representative Concentration Pathway 8.5 (RCP8.5) scenarios than under the Business-as-Usual (BAU) scenario (Fig. 1A). This pattern suggests that climate change and elevated CO_2_ concentrations are projected to enhance forest growth at a rate that surpasses increasing C losses via plant and soil respiration due to elevated air temperature. Because N deposition is higher under the BAU scenario due to relatively higher emissions, this result also suggests that the effects of increasing CO_2_ concentration and temperature outweigh the impacts of declining N deposition on ecosystem C sequestration. Total ecosystem C storage generally decreases with an increasing urbanization intensity, resulting in values being the highest under the RT scenario and the lowest under the GG scenario. Furthermore, the total ecosystem C storage under restrained urbanization and climate change (YC–RCP8.5 and RT–RCP8.5) scenarios exceed that of the Constant scenario, indicating that warmer climate with elevated CO_2_ concentrations promotes forest growth and counteracts the C loss resulting from moderate levels of urban expansion.

**Fig. 2.**
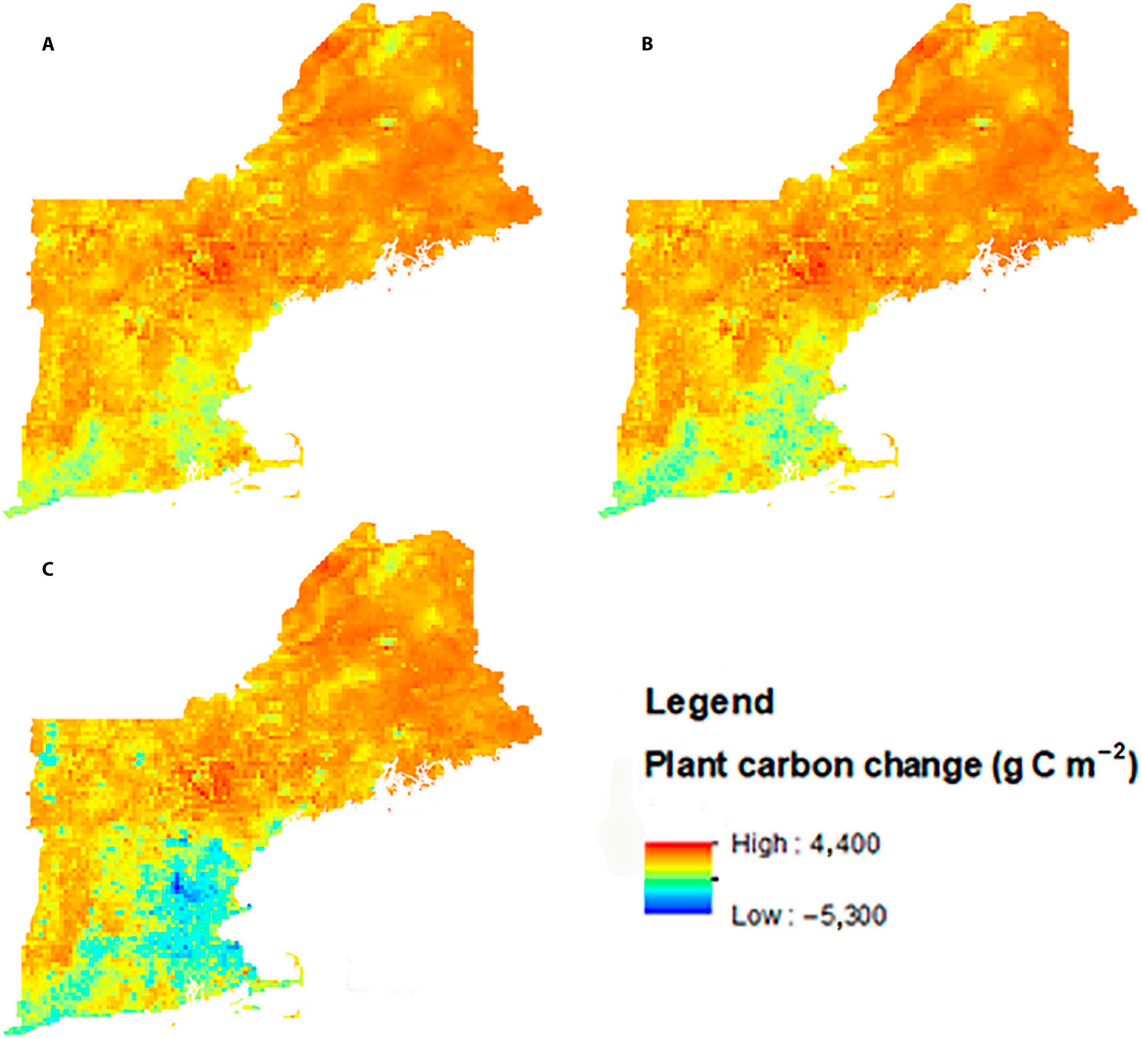
Spatial change in plant carbon storage (g C m^−2^) under Constant trend (A), Yankee Cosmopolitan (B), and Growing Global (C) future land-cover scenarios under the RCP8.5 and Clean Energy Futures (CES40B) scenario in 2050 compared to values in 2020.

Plant C storage is projected to continue to increase through the simulation period. The overall increase is primarily driven by climate change and CO_2_ fertilization, although the trend is partially offset by urbanization. Under a given air quality and land-cover scenario, plant C storage is greater under the RCP8.5 scenarios compared to that under the BAU scenario (Fig. 1B). Notably, land-cover change exerts a significant influence on the spatial patterns of plant C, resulting in greater C storage under moderate urban expansion (RT and YC scenarios) than under aggressive urban expansion (GG scenarios). This influence is more pronounced in southern New England than in the northern states (Fig. 2) and more distinct under scenarios with higher projected urbanization (i.e., GG). A distinct spatial pattern emerges, indicating net plant C loss in the areas near Boston and New Haven–Hartford–Springfield along with Burlington, VT, under the GG scenario (Fig. 2).

In general, SOM shows a declining trend throughout the simulation period, with lower SOM levels under the RCP8.5 scenarios than under the BAU scenarios for the same land-cover scenario (Fig. 2C) as projected temperature increases under RCP8.5 accelerate soil decomposition. Notably, SOM levels under the GG–BAU scenario are lower than those under the YC–RCP8.5 and RT–RCP8.5 scenarios, highlighting the strong negative effect of rapid urban expansion on SOM storage in the GG scenario.

### Future changes in N storage in New England under land-cover and climate change scenarios

**Fig. 3.**
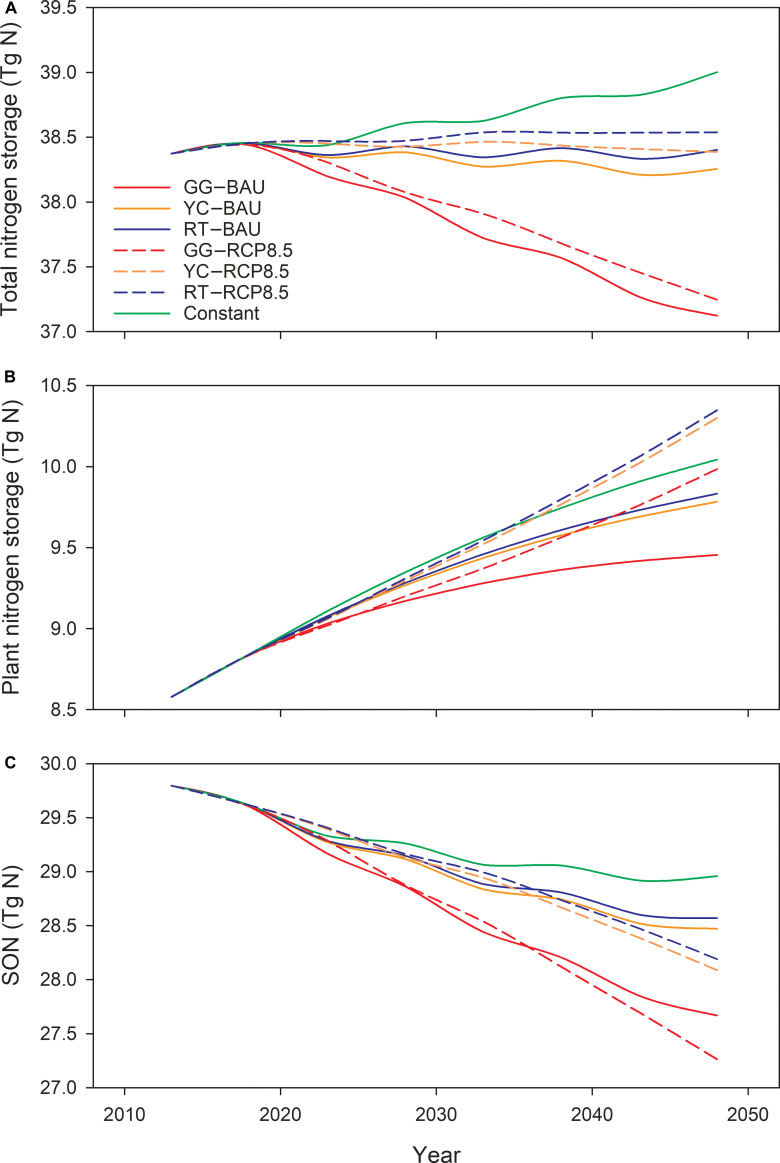
Time series of future projections of total ecosystem nitrogen storage (A), plant nitrogen storage (B), and soil organic nitrogen storage (C) for New England under different land-cover and climate scenarios from 2020 to 2050. SON, soil organic nitrogen.

The time-series patterns of N pools (Fig. 3) reveal consistent trends between plant N and C storage and between SON and SOM. This alignment reflects the relatively constant N concentration in plant tissues and SOM in model simulations. However, a distinctive pattern emerges in total ecosystem N storage (Fig. 3A), where the total ecosystem N storage for the entire New England region remains relatively stable under the YC and RT scenarios, with differences of less than 1% (Fig. 3A). In contrast, total ecosystem N consistently decreases under the GG scenarios, declining by an average of 3.2% from 240.8 g N·m^−2^ in 2020 to 237.5 g N·m^−2^ in 2050. By comparison, the total N storage under the Constant scenario is projected to increase by 4.7 g N·m^−2^ (2.0%), illustrating the positive impact of N deposition on total N storage and further demonstrating that atmospheric N deposition can help counterbalance N loss resulting from urbanization under the RT and YC scenarios. Notably, N storage under the RCP8.5 scenarios consistently surpasses that under the BAU scenarios across any given land-cover scenario, indicating the positive impact of climate change and elevated CO_2_ concentrations on N storage in forests.

**Fig. 4.**
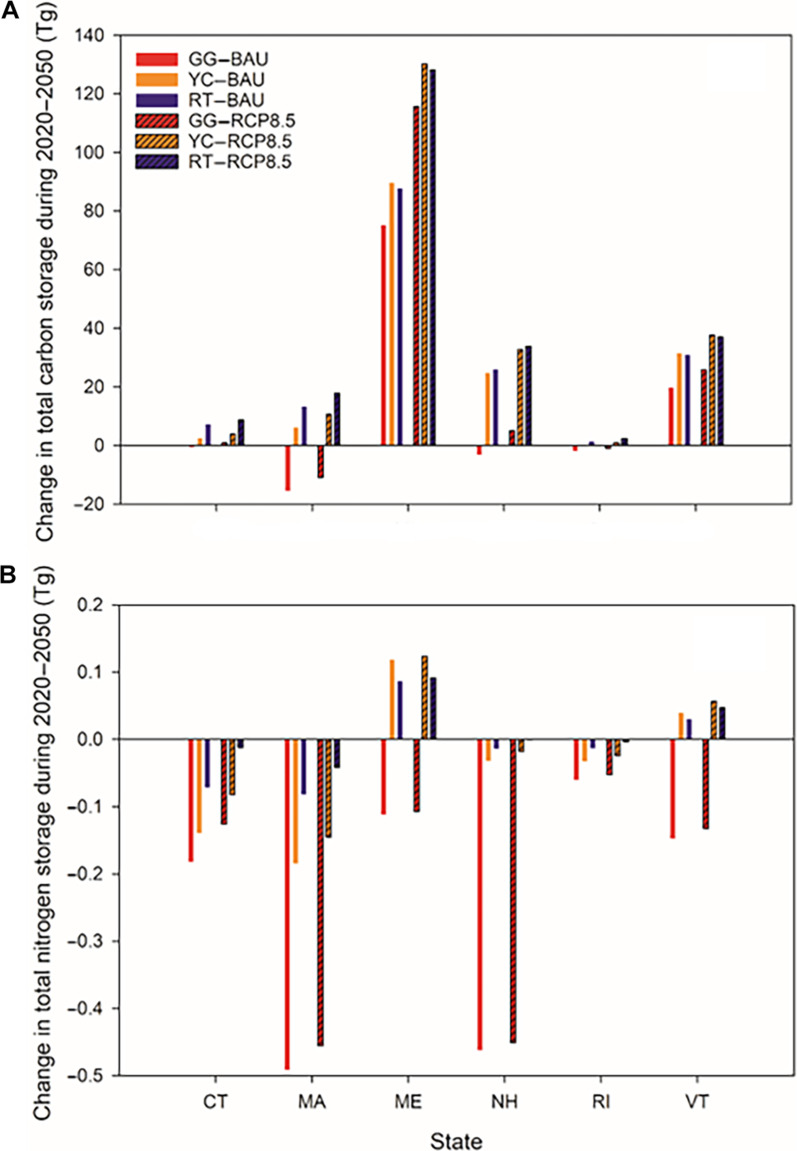
Changes in total ecosystem carbon storage (A) and nitrogen storage (B) for different states under future scenarios compared to those under the Constant scenario in New England from 2020 to 2050. CT, Connecticut; MA, Massachusetts; ME, Maine; NH, New Hampshire; RI, Rhode Island; VT, Vermont.

**Fig. 5.**
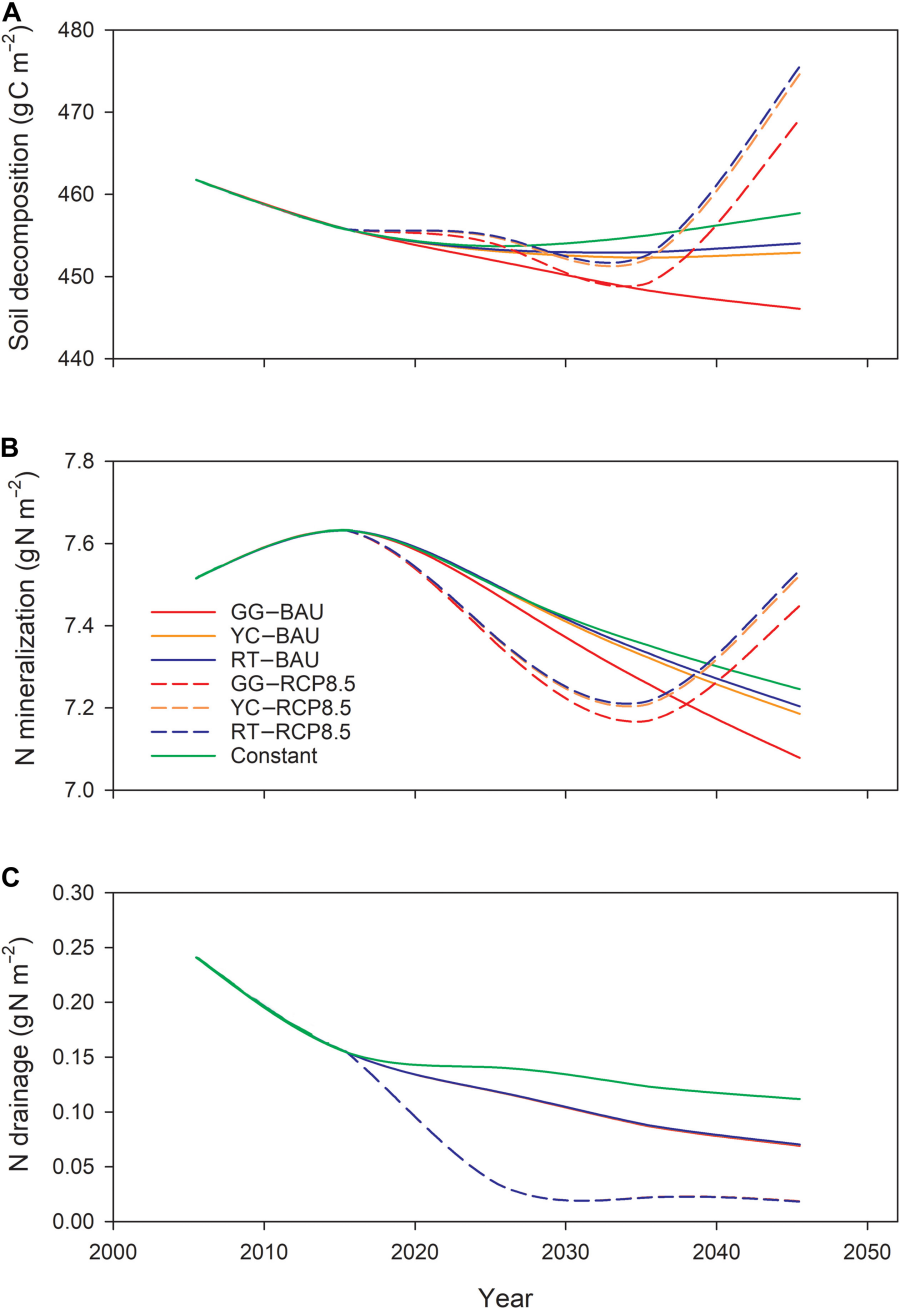
Time series of future projections of annual soil decomposition (A), annual N mineralization (B), and annual N drainage loss (C) for New England under different land-cover and climate scenarios from 2020 to 2050.

Changes in total ecosystem N storage are considerable among states, with projected N loss in CT, RI, MA, and NH under all land-cover scenarios compared to the Constant scenario and with N loss under BAU exceeding that under RCP8.5. However, total ecosystem N storage is projected to increase under YC and RT scenarios in ME and VT, while it decreases under GG scenarios (Fig. 4B). Additionally, ecosystem N accumulation under BAU is projected to be lower than that under RCP8.5 for a given land-cover scenario due to higher N leaching (Fig. 5C). MA is projected to experience the highest loss in total N storage, averaging a decline of 12.84 g N·m^−2^ (−5.86%) from 2020 to 2050 under the GG scenarios, followed by NH with a loss of 10.03 g N·m^−2^ (−3.78%) due to rapid urbanization in southern NH adjacent to Boston [1].

Future soil respiration and N mineralization initially decrease under the RCP8.5 scenario as a result of increasing urbanization, followed by a sharp increase after 2035 due to increasing temperatures (Fig. 5A and B). However, N drainage exhibited a contrasting pattern, initially decreasing and then remaining at a relatively low level (Fig. 5C). These patterns indicate higher plant N uptake due to higher forest growth under the RCP8.5 scenario, which helps limit N leaching and contributes to differences in total ecosystem N storage between the BAU and RCP8.5 scenarios. Notably, soil decomposition slightly increases after 2030, while net N mineralization continues to decline under the Constant scenario. This pattern indicates that increasing the soil C:N ratio suppresses N mineralization, further decreasing soil N availability.

### Future changes in water dynamics in New England under land-cover and climate change scenarios

Under the RCP8.5 scenario, annual precipitation in New England is projected to remain relatively stable, with a slight increase from 120.7 cm in 2020 to 122.0 cm in 2050. Snowpack is projected to decline, with an overall reduction of 59 cm from 2020 to 2050. ME is expected to experience the greatest decrease (89 cm), followed by CT (75 cm), NH (62 cm), MA (57 cm), and RI (51 cm). In contrast, VT is projected to experience a 38-cm increase. However, because southern New England states typically have shorter snow cover periods compared to the northern states, the impact of climate change on snowpack is more pronounced in the southern portion of the region. By 2050, snowpack levels in CT are projected to be only 32.4% of current levels; in RI, 23.6%; and in MA, 68.2%, whereas in northern New England, snowpack retention remains at approximately 95% of current levels. The duration of snow cover is also projected to decrease, with an average reduction of 11 d over the simulation period. ME experiences the largest decline (14 d), followed by CT and RI (13 d), MA (8 d), and NH and VT (7 d).

**Fig. 6.**
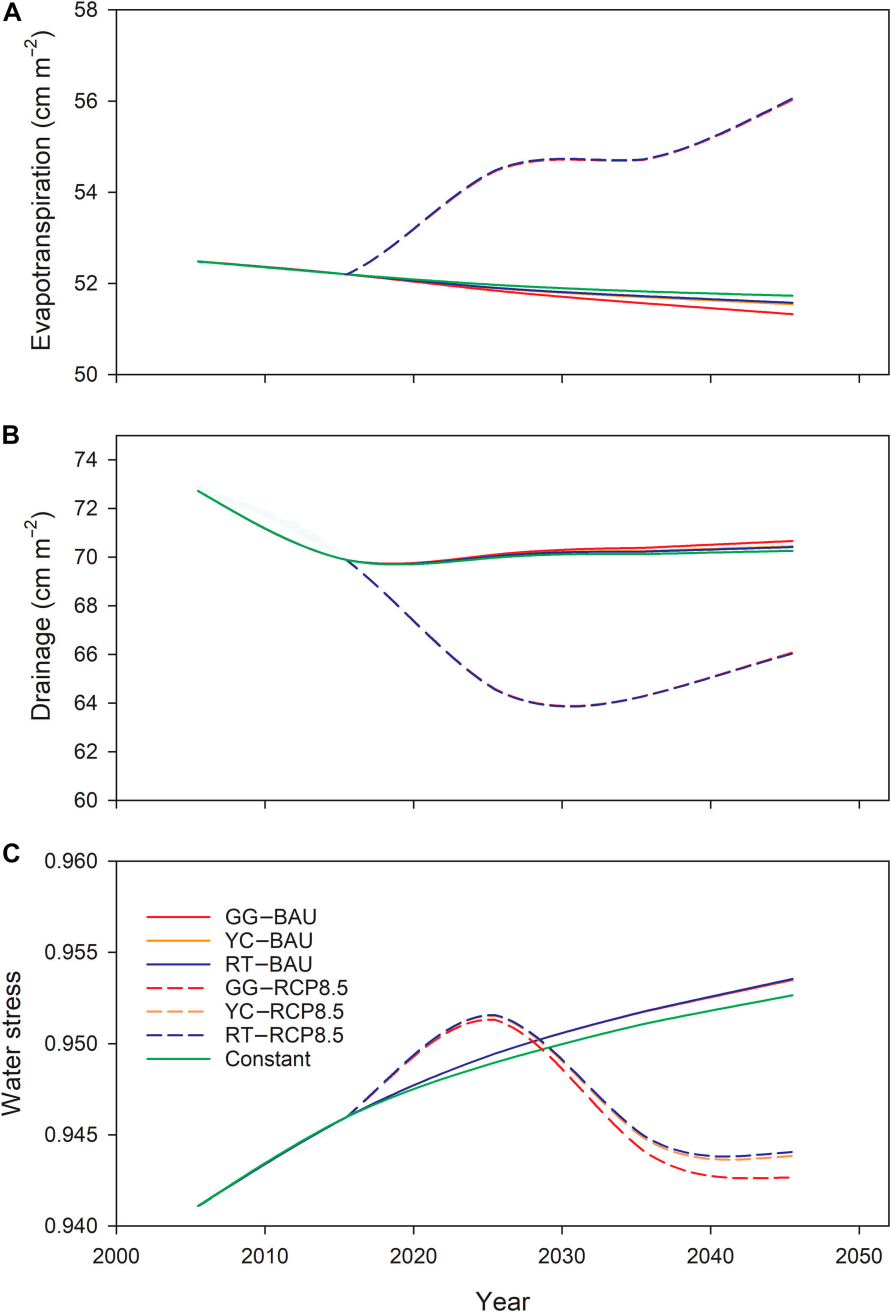
Time series of future projections of annual evapotranspiration (A), annual drainage (B), and deficit of water (Dwater) (C) for New England under different land-cover and climate scenarios from 2020 to 2050.

Distinct patterns of evapotranspiration (ET) also emerge across scenarios, with a slight decrease under the BAU scenarios but a general increase under the RCP8.5 scenarios (Fig. 6A). To better evaluate changes in soil water availability, we also examine deficit of water (Dwater), defined as the daily deviation of soil water supply from plant water demand. The projected decrease in forest area associated with urbanization leads to a decline in transpiration, contributing to decreases in ET and increases in drainage and Dwater under the BAU scenario (Fig. 6A and B). ET declines with increasing urbanization intensity and is lowest under the GG scenario due to extensive forest loss (Fig. 6A). Conversely, rising air temperatures under the RCP8.5 scenarios increase ET, which offsets the decline due to urbanization and continues over the simulation period. As a result, drainage decreases and soil water stress (Dwater) increases under the RCP8.5 scenarios (Fig. 6B). Notably, the average increase in photosynthetic rate under the RCP8.5 scenarios is 20.32%, far exceeding the increase in ET at 7.69% under the same scenarios. This disparity is driven by enhanced WUE resulting from elevated CO_2_ concentrations.

## Discussion

### Future changes in carbon storage in regional simulations in New England

In this study, we project that the forests of New England will continue to serve as a dominant C reservoir for at least the next 3 decades. The main projected increase in C storage is attributed to the increase in plant C storage, aligning with previous modeling studies for the region [19–22]. The annual patterns of total ecosystem C storage show that under the same air quality and land-cover scenarios, C storage is greater under the RCP8.5 scenarios compared to that under the BAU scenarios (Fig. 1A), highlighting the effects of CO_2_ fertilization and climate change on forest growth.

Although elevated CO_2_ concentrations and atmospheric N deposition in urban areas may stimulate forest growth and increase total and plant C storage, we project that plant C storage will decrease in urban areas, and this effect will become more evident with increasing urbanization (Fig. 2). This result suggests that forest removal due to urbanization has a more significant impact on forest biomass than the effects of climate change and air quality changes. We found that CO_2_ concentration increases by only 0.16 ppm with a 1% increase in urbanization in New England [17]. Therefore, this limited enrichment of CO_2_ across the urbanization gradient suggests that the atmospheric CO_2_ fertilization effect on forest growth will be limited in urban and suburban areas of New England.

Our previous modeling studies at Harvard Forest suggest that the atmospheric CO_2_ fertilization effect is a more important driver than the effects of temperature on recent plant C accumulation [17,23]. In most modeling studies predicting C change in the northeastern USA, the atmospheric CO_2_ fertilization effect results in a positive response of forest growth under a warming climate [19–21]. However, without invoking this mechanism, some analyses project a neutral or negative tree growth response to a warming climate [19]. However, a few studies suggest positive forest responses to a warming climate without atmospheric CO_2_ fertilization [21,24]. Nevertheless, our previous analysis also suggests that atmospheric CO_2_ fertilization effects will ultimately be limited by nutrient availability. We projected future changes in total ecosystem C storage over an 80-year period under different climate scenarios at Harvard Forest and found no significant difference under the Representative Concentration Pathway 4.5 (RCP4.5) and RCP8.5 scenarios [23], consistent with observations from free air CO_2_ enrichment experiments, which show that CO_2_ enrichment declines and ultimately disappears after years of experimentation [25].

### Future changes in N storage in regional simulations

The spatial patterns of total N storage in New England differ from the patterns of total C storage due to the predominant storage of N in soil, which is significantly influenced by land-cover changes. The temporal trend observed under the Constant scenario suggests an increase in total ecosystem N storage over the simulation period in the absence of land-cover change. However, this upward trajectory is offset by moderate urbanization and reverses to a downward trajectory with aggressive urbanization. Notably, the projected total N storage for CT, MA, RI, and NH under all land-cover scenarios is lower than the values under the Constant scenario (Fig. 4B), reflecting substantial N loss due to urbanization.

With elevated plant growth and decreasing N deposition, the rate of plant N uptake exceeds the natural replenishment rate of soil N, leading to a decline in soil N storage. Elevated CO_2_ concentrations [26] and temperature [27,28] contribute to increased soil decomposition, further decreasing N content in the soil (Fig. 3C). The decline in soil N storage reflects ongoing N oligotrophication of forest ecosystems in rural areas of New England [29–31]. Trends of N oligotrophication have been observed in temperate forests in recent years, evident in indicators such as declining nitrate leaching and natural abundance foliar ^15^N values. Long-term observations from the Hubbard Brook Experimental Forest in NH support this trend, illustrating a decline in N availability to trees and an increase in N immobilization [30].

In our projections, N oligotrophication appears in the relationship between soil decomposition and N mineralization under the RCP8.5 scenarios (Fig. 5A and B). Increases in soil decomposition substantially exceed increases in N mineralization after 2030, indicating a net decrease in N release per unit of SOM mineralization. Additionally, a decline in N drainage is evident in model projections and is more pronounced under the RCP8.5 scenarios than under the BAU scenarios (Fig. 6C). Finally, N deposition is expected to decrease in New England due to controls on emissions from electric utilities and transportation sources [32], which will likely further intensify N oligotrophication in the region if there is no further change in fossil fuel or agricultural emissions.

### Future changes in water dynamics in regional simulations

Urbanization is expected to increase water drainage [33], which is observed under the BAU scenario but not under the RCP8.5 scenario. This phenomenon can be attributed to ET increases under the RCP8.5 scenarios due to elevated temperatures, even with a concurrent increase in WUE resulting from elevated CO_2_ concentrations [14]. Elevated ET enhances soil water loss, allowing increases in precipitation to replenish soil water rather than being exported as runoff.

Climate change is projected to cause a decline in snow days and snowpack across the entire region, with more pronounced impacts in southern New England than in northern areas. This reduction is likely to negatively affect winter recreation, particularly ski resorts in southern New England. By 2050, snowpack levels are projected to decrease to 32.4% of current levels in CT, 23.6% in RI, and 68.2% in MA. Additionally, fewer snow days are projected to lead to increased winter streamflow and reduced spring snowmelt discharge [13].

### Model validation and future study

Simulated AGB is slightly higher than observed values in coastal regions but lower in the White and Green mountains. The coastal overestimation may stem from the limited representation in the model of climate-related disturbances, such as high-wind events, and the moderating effects of coastal climates on forest growth. In contrast, the underestimation of AGB in the White and Green mountains (Fig. S1) is consistent with the tendency of models to underestimate forest growth at higher elevations. A likely reason for this underestimation is that N availability may be higher at high elevations, contrary to patterns in model simulations. Fahey et al. [34] found that N mineralization rates and foliar N concentrations increase with elevation at the Hubbard Brook Experimental Forest, whereas the model predicts a decreasing trend. However, the mechanisms underlying enhanced N mineralization at higher elevations remain poorly understood and warrant further investigation.

Simulated SOM tends to be higher than observed values in northern regions, consistent with the pattern of increasing bias with decreasing deciduous fraction and increasing elevation. The trend along the deciduous fraction gradient likely arises because lower deciduous fractions are associated with conifer-dominated forests in colder northern areas. This discrepancy may stem from how the model represents SOM decomposition—accounting only for the aerobic soil decomposition pathway while neglecting natural pathways such as the microbial anaerobic pathway or erosion. Furthermore, the SOM bias is particularly pronounced at elevations above 800 m, which may reflect the absence of key SOM removal mechanisms in the model, such as erosion, enhanced decomposition, and dissolved organic carbon leaching [34], or recovery from acid deposition [35], all of which are more prominent in high-altitude environments. The increasing bias along the ISA gradient is partly due to differences in how SOM is represented: in the model, SOM values are averaged over areas using ISA-weighted means, whereas observational data are directly derived from site-specific soil sampling. Moreover, our algorithms to characterize and quantify the effects of urbanization may underestimate the actual effects, especially water stress on forest growth [2].

We aim to apply our model in other regions, but substantial uncertainties remain, and further research is needed to refine these projections. A primary limitation is that air quality is highly dependent on local energy use and urbanization rate. For example, the Beijing–Tianjin–Hebei region of China may experience dynamics quite different from those of New England due to higher population and emissions of NO and volatile organic compounds from coal combustion, whereas New England relies more on natural gas. Urbanization further intensifies pollutant concentrations, leading to more severe O_3_ damage to plants and suppressing photosynthesis and growth. With continued air pollution controls, C accumulation in such regions could increase as ozone-related damage declines [36]. At the same time, reductions in N deposition may drive N limitation (oligotrophication) over time, a phenomenon observed in New England. In addition, vegetation type plays a critical role. PnET has been extensively applied to northeastern US forests for decades. Before extending the model to other regions, stand-level testing for the dominant vegetation types in the target region is necessary to ensure model reliability.

The PnET model will require continued development. Emerging research highlights ecological mechanisms not yet incorporated into terrestrial models, potentially contributing to underestimation of temperature effects on carbon dynamics. For instance, Meng et al. [37] reported that foliar C allocation increases in the early green-up period driven by climate change, a process not represented in the PnET model or other terrestrial ecosystem models. Such mechanisms highlight important gaps in current modeling frameworks and warrant further investigation.

## Conclusion

In this study, we projected potential changes in forest ecosystem function under various land-cover and climate change scenarios. Model calibration results indicate that AGB is reasonably well simulated at the regional scale, although the model tends to overestimate SOM, particularly in northern and high-elevation mountainous areas. These biases highlight areas where model performance could be improved in future studies, especially by incorporating additional SOM decomposition pathways and better representing elevation-driven processes.

Future projections indicate that New England will continue to serve as a regional C sink, but important trade-offs emerge from land-cover changes impacting C sequestration, especially in southern New England. Urbanization will reduce C storage, especially in soil C pools. In addition, climate change is projected to facilitate increases in soil decomposition, further intensifying soil C loss. The role of atmospheric CO_2_ in future forest response remains critical; however, due to developing nutrient limitations, the fertilization effect is likely to be weaker than previous studies have projected.

Urbanization and climate change act together to limit N accumulation in New England. Extreme land-cover change associated with urbanization substantially reduces N storage. With continuous N accumulation in plants, soil N storage declines, reducing soil N availability and driving ecosystems toward a condition of N oligotrophication. N oligotrophication is projected to curtail forest growth in rural areas and will become more pronounced with decreasing N deposition due to air quality emission controls. This phenomenon will need to be considered in future management of northeastern forests.

Even though previous studies indicate that the New England region will experience hotter and wetter weather, our study shows that water stress will not significantly increase. In addition, we found that snowpack and the duration of snow cover will significantly decrease in southern New England, potentially altering water dynamics in that part of the region.

## Methods

### Study region and calibration sites

New England covers an area of 162,716 km^2^ in the northeastern USA encompassing the states of ME, VT, NH, MA, CT, and RI. Notably, 80.1% of this region is forest, 8.6% is developed land, and 6.4% is agricultural land [1]. The climate of New England is a humid continental climate, featuring an annual mean temperature ranging from 3 to 10 °C and an average annual precipitation ranging from 790 to 2,550 mm [38]. This range of climatic conditions leads to distinct vegetation types, with the forest transitioning from boreal forests in northern ME to oak–maple forests in southern New England [39].

To calibrate the PnET-CN-daily model for hardwood forests and boreal forests, we utilized data from Harvard Forest, MA, and Howland Forest, ME, respectively. Harvard Forest is in central MA (42°28′N, 72°10′W). It has served as a National Science Foundation (NSF)-supported Long Term Ecological Research site since 1988, with weather records from 2002 to 2019 revealing an annual average temperature of 8.3 °C and an annual precipitation of 1,254 mm. The forest in this area is classified as a temperate mixed forest, with northern red oak (*Quercus rubra*), eastern hemlock (*Tsuga canadensis*), white pine (*Pinus strobus*), red maple (*Acer rubrum*), and black birch (*Betula lenta*) [40].

Howland Forest is in central ME (45°12′N, 68°44′W) and has been a research site since 1986. It is located within a transition zone between the boreal forest to the north and the northern hardwood forest to the south. The forest composition is primarily dominated by red spruce (*Picea rubens*) and hemlock, with hardwood species, mainly red maple and paper birch (*Betula papyrifera*), collectively accounting for less than 10% of the total basal area [41].

### Model description and improvement

The PnET model is a process-based ecosystem model that simulates C, N, and water dynamics in forest ecosystems at a monthly time step. In this study, we utilized the PnET-CN-daily model, which represents an advancement of the PnET-CN model and offers 2 significant advantages over previous versions. First, it simulates a more complete N cycle in soil, including processes such as net N mineralization, nitrification, plant N uptake, and leaching losses, a feature that was lacking in the earlier PnET-II model and subsequent versions. Second, PnET-CN-daily operates at a daily time step, enhancing the precision of simulation of plant phenology and water dynamics. Our prior work demonstrated that the PnET-CN-daily model exhibits notable improvements over earlier PnET models in simulating biogeochemical processes at Harvard Forest, particularly in capturing the increasing trends in GPP associated with climate change and the impacts of water stress [23].

To employ the PnET-CN-daily model, several inputs are needed, including site characteristics, plant traits, and various environmental data. The environmental data include daily maximum and minimum air temperatures, photosynthetically active radiation (PAR), and precipitation as meteorological inputs, as well as atmospheric concentrations of CO_2_ and O_3_ and atmospheric N deposition (NH_4_^+^ and NO_3_^−^). The site characteristics and plant traits employed in this study were adopted from Ollinger et al. [42] and were adjusted during the model calibration.

To evaluate soil water stress, the PnET models use Dwater to measure soil moisture, which is defined as follows:

$$\text{Dwater}=\frac{\sum_{i=1}^{n}\frac{{\text{Trans}}_{i}}{{\text{PotTrans}}_{i}}}{n}$$ (1)

where *Trans_i_* is the simulated transpiration on the ith day, ${\text{PotTrans}}_{i}$ is the theoretical transpiration without considering any restriction in the ith day, and *n* is the number of days when ${\text{Trans}}_{i}$ is less than ${\text{PotTrans}}_{i}$ in the period. The maximum value of Dwater is 1, which indicates no water stress, and soil water stress increases with decreasing Dwater.

### Future scenarios’ construction

In this study, we applied the PnET-CN-daily model to simulate forest ecosystem dynamics from the year 1000 to 2050. A reconstruction of historical climate and air quality scenario from 1000 to 2020 was conducted for Harvard Forest in our previous study [17], and we extended this approach to generate a regional-scale historical dataset for New England.

In this study, we considered a suite of future scenarios from 2020 to 2050 that depict potential future changes in land cover, climate, and air chemistry. To represent potential future land-cover change, we used 3 scenarios developed by the New England Landscape Futures (NELF) project: RT, YC, and GG [1]. The RT scenario assumes that urbanization will continue at the average rate and spatial pattern observed between 1990 and 2015, serving as a reference scenario. The GG and YC scenarios were co-designed with >200 informed stakeholders from throughout the region [22]. The GG scenario represents an aggressive urbanization scenario, where a substantial influx of climate migrants drives significant population growth, resulting in extensive conversion of forest to urban land. The YC scenario also assumes a substantial influx of migrants into the region but incorporates technological advances to enhance resilience to climate change and accommodate the increasing population. In this scenario, a substantial area of forest is retained compared to that in the GG scenario. In addition to the New England Landscape Futures scenarios, we also developed a Constant scenario, in which urbanization remains at current levels, as a static reference.

Two future climate scenarios involved changes in air temperature, precipitation, and PAR. The RCP8.5 was derived from the Climate Explorer project (https://crt-climate-explorer.nemac.org/). The Climate Explorer employs 2 potential emission scenarios (RCP4.5 and RCP8.5) in conjunction with a set of global climate models to predict future climate conditions. The result is a weighted mean of the outputs from 32 climate models in the Localized Constructed Analogs method dataset. PAR was calculated using the MTCLIM model based on air temperature and precipitation data. We also created a BAU scenario in which the measured temperature and precipitation values from 2011 to 2020 are repeated for the period from 2021 to 2050.

Future scenarios for O_3_ concentration and N deposition were obtained from the Clean Energy Futures project [32]. In this project, the Integrated Planning Model and Community Multiscale Air Quality model were executed in series with various scenarios for the decarbonization of the electricity sector to forecast future air quality [32]. We used the model outputs for the BAU scenario (C1) and an aggressive decarbonization scenario (CES40B) to assess the potential effects of changes in air quality in New England resulting from the decarbonization of the electricity sector. The CES40B scenario assumes the complete elimination of emissions from fossil fuel combustion in electrical generating units by 2040 [32].

To calculate the ISA for New England, land-use information was obtained from the National Land Cover Database 2016 release (https://www.usgs.gov/centers/eros/science/annual-nlcd-data-access). These data are available at a 30-m resolution, and each pixel is categorized into different land-cover types, including water surface, developed land, and vegetation. Developed land is further classified into 4 categories based on the ISA percentage of land cover: less than 20%, 20% to 49%, 50% to 79%, and 80% to 100%. To simplify the data, we assigned ISA values of 10%, 35%, 65%, and 90% for these classes, respectively. The percentage of ISA for a specific *x*-km-resolution pixel is calculated as

$$\%\mathrm{ISA}=\frac{\sum_{n}i}{N}\times 100\%$$ (2)

where %ISA is the ISA percentage in a pixel of a given resolution (*x* km), *n* is the number of pixels with impervious surface cover, *i* is the ISA percentage of pixels with the impervious surface cover, and *N* is the number of total pixels within the *x*-km-resolution pixel.

We combined the 3 land-cover scenarios (GG, YC, and RT) with 2 climate scenarios (BAU and RCP8.5) and 2 air quality scenarios (C1 and CES40B) to create a series of future scenarios for application across New England. For areas projected to experience future urbanization, we assume that land cover will transition from its current state to the projected 2050 land-cover scenarios at a constant rate. Air temperature, atmospheric CO_2_, ground-level O_3_, and atmospheric N deposition in urban areas were adjusted based on local %ISA using empirical relationships between these factors and %ISA for New England developed previously [23].

### Model calibration

To assess the accuracy of PnET-CN-daily model simulations, we conducted model runs using reconstructed historical scenarios for Harvard Forest and Howland Forest. We compared the model outputs with observed data collected at these sites. Model calibration for the hardwood forest at Harvard Forest was based on several ecosystem variables documented in our previous study [17]. To calibrate model performance in the conifer forest, observations from the Howland Forest AmeriFlux site [43] including GPP, net ecosystem production, respiration, wood mass, fine root mass, SOM, and SON were used for calibration.

In addition to site calibration, we conducted a regional evaluation for both AGB and SOM. For AGB calibration, we utilized the contiguous US biomass map generated by the US Department of Agriculture (USDA) Forest Service Forest Inventory and Analysis (FIA) program, which includes observations at a 250 × 250 m resolution. We sourced SOM data from the Gridded National Soil Survey Geographic (gNATSGO) database, a USDA compilation amalgamating various soil databases. This database integrates information from the Soil Survey Geographic Database, State Soil Geographic Database, and Raster Soil Survey Databases, presenting a cohesive ESRI file geodatabase with a resolution of 30 × 30 m. To compare regional data with the model outputs, we aggregated the gNATSGO and FIA data to a 4 × 4 km resolution that was used as the spatial resolution for simulations.

In addition to visual evaluation, PBIAS, normalized mean error (NME), and NMAE were used to evaluate model calibration. These parameters enabled assessment of the ability of model simulations to capture average and long-term trends in observed values and are defined as follows:

$$\text{PBIAS}=100\times \frac{P_{i}-O_{i}}{O_{i}}$$ (3)

$$\mathrm{NME}=\frac{\bar{P}-\bar{O}}{\bar{O}}$$ (4)

$$\text{NMAE}=\frac{\sum_{i=1}^{n}\left(\left|P_{i}-O_{i}\right|\right)}{n\times \bar{O}}$$ (5)

where $P_{i}$ is the ith predicted value, $O_{i}$ is the ith observed value, $\bar{P}$ is the mean of the predicted value, $\bar{O}$ is the mean of the observation, and n is the number of observations.

PBIAS is used to measure the percentage difference between simulation and observation. NMAE is used to evaluate model performance in capturing observed trends, where lower NAME values indicate better model agreement with observations. NME is used to determine whether the model overestimates or underestimates parameters. A negative value indicates underestimation, and a positive value shows overestimation relative to observations.

## Data Availability

The dataset presented in this article is available for reviewers on Dryad at http://datadryad.org/share/K4AgIHLGm8VprdrLgpAA-3YIKjta50kPg3HB73z06yQ and will be made publicly available upon publication through both Dryad and the Harvard Forest Data Archive.

## Supplementary Materials

Supplementary 1 Figs. S1 and S2 Tables S1 to S4 Reference [44]
