# Supplementary material for: Regional Projections of the Impacts of Future Urbanization and Climate Change on Biogeochemical Cycles in New England Landscapes
Source: Research (Wash D C). 2025 Dec 16;8:1043. doi: 10.34133/research.1043 (PMC12705937; doi:10.34133/research.1043)
Supplement: Supplementary 1 — Figs. S1 and S2 Tables S1 to S4 Reference [44] [file research.1043.f1.docx]

# SUPPLEMENTARY MATERIALS

**Contents**

Description of PnET-CN-daily model regional calibration

Figure S1. Spatial distribution of percent bias in aboveground biomass (AGB) across New England

Figure S2. Spatial distribution of percent bias in soil organic matter (SOM) across New England.

Table S1. Comparison of model-calculated ecosystem fluxes and pools of carbon and nitrogen with measured values at Howland Forest.

Table S2. Aboveground biomass (AGB) and soil organic matter (SOM) simulation performance stratified by elevation for New England.

Table S3. Aboveground biomass (AGB) and soil organic matter (SOM) simulation performance stratified by an impervious surface area (ISA) gradient in New England.

Table S4. Aboveground biomass (AGB) and soil organic matter (SOM) simulation performance stratified by deciduous fraction of forest cover for New England.

**Description of PnET-CN-daily model regional calibration**

The overall average simulated AGB is 10,232 ± 1634 g C∙m⁻², compared to an observed average of 9255 ± 5149 gC∙m⁻². The NAE is 0.11, and the NMAE is 0.43, indicating the model slightly overestimates AGB at the regional scale with a moderate level of deviation. Spatial patterns of percentage difference between simulation and observation reveal that overestimation is particularly evident in southern Maine and southeastern Massachusetts. This may be due to the model overestimating climate impacts on those coastal areas. Conversely, model underestimation is pronounced in the White and Green Mountains of New Hampshire and Vermont, respectively, possibly reflecting an overestimation of natural disturbance on forest structure or the model failure to simulate recovery from acid rain impacts which have been evident in high elevation forests in this area [44].

Stratifying the results by elevation shows that more than 48% of the region lies below 200 m, where the model performs well, with a bias of less than 2%. At higher elevations, observed AGB values generally exceed simulated values, and the difference increases with elevation. This is primarily because the model simulates a decline in AGB with increasing elevation, whereas observations indicate an upward trend, with AGB peaking between 800 and 1000 m before declining. When stratifying by impervious surface area (ISA), approximately 90% of New England falls within the 0–10% ISA range, where both bias and percentage difference are low. Although the absolute bias increases to over 5000 gC∙m⁻² in areas with higher ISA, the percentage difference remains low and even decreases as ISA increases. Examining AGB by deciduous forest ratio reveals that observed AGB values are generally higher with increasing deciduous cover, peaking in the 60–80% range. Across most deciduous cover classes, the model slightly underestimates AGB, with relatively small percentage differences. However, extreme deviations occur in areas with very low (<20%) or very high (>80%) deciduous cover (Table S3) likely corresponding to regions in southern Maine and southeastern Massachusetts. These outliers contribute disproportionately to the total difference between simulated and observed AGB.

The overall average simulated SOM is 16682 ± 3569 gC∙m⁻², compared to an observed average of 14178 ± 6110 gC∙m⁻². The NAE is 0.17, and the NMAE is 0.40, indicating the model slightly overestimates SOM at the regional scale with a moderate level of deviation. Spatial patterns of percentage difference between simulation and observation reveal that overestimation is particularly evident in northern regions, while underestimating it in the southern urban areas. This discrepancy may occur because the model accounts only for SOM loss through anthropogenic decomposition while neglecting other decomposition pathways, leading to an overestimation of SOM accumulation. Additionally, the model simulates SOM accumulation starting from the year 1000, whereas the actual historical scenario is likely more complex than these model assumptions. Finally, recent observations suggest SOM loss from high elevation forests in New Hampshire may be a manifestation of recovery of soil decomposition process from effects of chronic acidic deposition (Possinger et al. 2025).

When stratifying by ISA, approximately 90% of New England falls within the 0–10% ISA range and model generally overestimate where both bias and percentage difference are relatively high mainly due to northern forest region. The model performance is the best with ISA range from 10 to 30%, where in the southern low urbanized region. However, with ISA further increase, the model is likely to underestimate SOM. The difference is a result of the way how to represent the SOM, in our model study, we averaged SOM value by weighted ISA ratio, while observations we use is soil sampling result.

Stratifying the results by elevation shows that both bias and percentage difference in SOM increase significantly at higher elevations (>800 m). Observations indicate that SOM peaks between 600 and 800 m and then declines, whereas the model continues to simulate increasing SOM with elevation. At these higher elevations, observed AGB values also tend to exceed simulated values, with the difference widening as elevation increases. While the model successfully captures the decreasing trend of AGB at high elevations, it fails to reproduce the observed decline in SOM. This discrepancy may be due to missing mechanisms of SOM removal at high elevations, such as erosion, leaching, higher decomposition, or recovery from acid deposition.

When examining SOM by deciduous forest ratio, the model generally underestimates SOM in areas with high deciduous cover (around 80%) but tends to overestimate SOM as the deciduous ratio decreases. Since high deciduous cover typically occurs in northern areas, this pattern suggests that the model may be overestimating SOM in colder, high elevation regions.


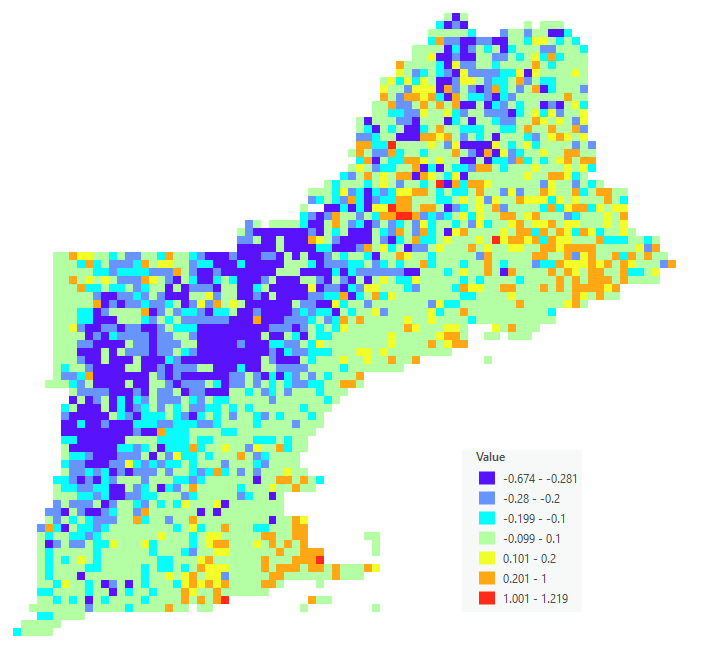


Figure S1. Spatial distribution of percent difference in aboveground biomass (AGB) across New England.


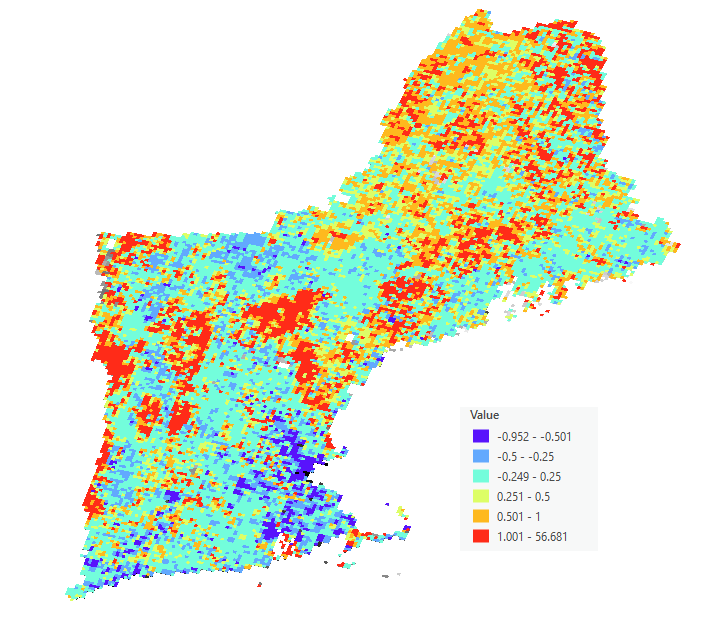


Figure S2. Spatial distribution of percent difference in soil organic matter (SOM) across New England.

Table S1. Comparison of model-calculated ecosystem fluxes and pools of carbon and nitrogen with measured values at Howland Forest.

| Parameter | Period | Observation | Simulation | NMAE | NAE |
| --- | --- | --- | --- | --- | --- |
| GPP^*^ | 1996-2020 | 1324.94 ± 83.97 | 1350.73 ± 91.85 | 0.09 | 0.02 |
| NEP^*^ | 1996-2020 | 210.52 ± 39.95 | 183.53 ± 61.77 | 0.21 | -0.12 |
| Respiration^*^ | 1996-2020 | 1114.44 ± 96.13 | 1167.2 ± 88.22 | 0.12 | 0.05 |
| Wood mass^**^ | 1987 | 9297.26 ± 1137.89 | 9274.72 ± 598.913 | 0.05 | -0.002 |
| Fine root^**^ | 1987 | 810 | 796.86 |  |  |
| SOM^**^ | 1987 | 11000 | 10207.3 |  |  |
| SON^***^ | 1987 | 330.9 | 316.8 |  |  |

* The unit is gC·m^-2^·year^-1^

** The unit is gC·m^-2^

*** The unit is gN·m^-2^

Table S2. Aboveground biomass (AGB) and soil organic matter (SOM) simulation performance stratified by elevation for New England.

| Elevation  (m) | 0-200 | 200-400 | 400-600 | 600-800 | 800-1000 | >1000 |
| --- | --- | --- | --- | --- | --- | --- |
| AGB Simulation | 10678 ± 1778 | 9938 ± 1304 | 9695 ± 1228 | 9042 ± 1029 | 8342 ± 908 | 8302 ± 941 |
| AGB Observation | 7340 ± 5259 | 10296 ± 4403 | 11966 ± 3615 | 13293 ± 3447 | 14425 ± 2005 | 11682 ± 4951 |
| AGB Bias | 3370 ± 5356 | -362 ± 4293 | -2264 ± 3428 | -4204 ± 3530 | -6082 ± 1768 | -3380 ± 5645 |
| Percent Bias in AGB | 1.97±17.17 | -9.19±20.16 | -17.31±21.42 | -30.50±15.08 | -38.40±14.00 | -26.65±47.03 |
| SOM Simulation | 15637 ± 4101 | 17985 ± 2665 | 17242 ± 2058 | 17496 ± 2508 | 20002 ± 2770 | 23316 ± 1752 |
| SOM Observation | 13647 ± 6200 | 14326 ± 5690 | 15587 ± 5936 | 16242 ± 6692 | 13590 ± 6824 | 8883 ± 4215 |
| SOM Bias | 1971 ± 6817 | 3659 ± 6250 | 1655 ± 6501 | 1244 ± 7175 | 6412 ± 7947 | 14433 ± 4467 |
| Percent Bias in SOM | 34.65±73.48 | 46.59±70.94 | 28.30±56.65 | 27.89±59.95 | 81.89±88.96 | 188.67±83.26 |
| Number | 3279 | 2131 | 1041 | 275 | 49 | 9 |

* The units of simulation, observation, and bias are in gC·m⁻². The unit of percent bias is %.

Table S3. Aboveground biomass (AGB) and soil organic matter (SOM) simulation performance stratified by ISA gradient for New England.

| ISA (%) | 0 – 10 | 10 – 20 | 20 – 30 | 30 – 40 | 40 – 50 | >50 | Total |
| --- | --- | --- | --- | --- | --- | --- | --- |
| AGB Simulation | 10261 ± 1469 | 10808 ± 1702 | 9579 ± 1748 | 8158 ± 1920 | 7303 ± 2833 | 4424 ± 2629 | 10232 ± 1634 |
| AGB Observation | 9844 ± 4726 | 5265 ± 5831 | 2854 ± 4993 | 1923 ± 4359 | 1524 ± 3836 | 504 ±2123 | 9956 ± 5149 |
| AGB  Bias | 426 ± 5050 | 5711 ± 5912 | 6946 ± 4954 | 6119 ± 4568 | 5459 ± 4443 | 3920 ± 3456 | 976 ± 5361 |
| Percent Bias AGB | -6.84 ± 21.25 | 1.74 ± 18.76 | -0.45 ± 13.92 | -1.58 ± 11.87 | -3.56 ± 13.48 | -1.14 ± 15.58 | -7.66 ± 23.54 |
| SOM  Simulation | 17517 ± 2548 | 11428 ± 2060 | 8709 ± 1714 | 6664 ± 2041 | 5365 ± 3054 | 2550 ± 1646 | 16682 ± 3569 |
| SOM  Observation | 14444 ± 5992 | 13104 ± 6382 | 11903 ± 6421 | 12756 ± 6902 | 11203 ± 6356 | 10351 ± 8291 | 14177 ± 6110 |
| SOM  Bias | 3065 ± 6396 | -1675 ± 6400 | -3282 ± 6273 | -6091 ± 6873 | -5837 ± 7070 | -7802 ± 8169 | 2445 ± 6675 |
| Percent Bias in SOM | 42.28 ± 69.29 | 6.67 ± 59.51 | -3.21 ± 78.62 | -24.15 ± 56.31 | -32.34 ± 54.00 | -54.17 ± 95.62 | 38.51 ± 89.94 |
| Number | 6145 | 384 | 139 | 78 | 38 | 36 | 6820 |

* The units of simulation, observation, and bias are in gC·m⁻². The unit of percent bias is %.

Table S4. Aboveground biomass (AGB) and soil organic matter (SOM) simulation performance stratified by deciduous fraction of forest cover for New England.

| Deciduous Fraction | 0 – 20 | 20 – 40 | 40 – 60 | 60 – 80 | > 80 |
| --- | --- | --- | --- | --- | --- |
| N | 326 | 1388 | 2538 | 1678 | 842 |
| AGB Simulation | 9998 ± 1963 | 9845 ± 1461 | 10101 ± 1342 | 10402 ± 1573 | 10792 ± 2190 |
| AGB  Observation | 6906 ± 4521 | 8693 ± 4277 | 9590 ± 4714 | 10246 ± 5336 | 8373 ± 6490 |
| AGB Bias | 3030 ± 5488 | 1153 ± 4725 | 549 ± 4874 | 174 ± 5641 | 2437 ± 6251 |
| Percent Bias in AGB | -6.04 ± 25.24 | 0.86 ± 23.22 | -7.07 ± 19.69 | -11.19 ± 20.21 | -6.82 ± 16.45 |
| SOM Simulation | 21521 ± 2938 | 19608 ± 2171 | 17418 ± 2064 | 15213 ± 2417 | 11506 ±3275 |
| SOM  Observation | 14521 ± 6382 | 14590 ± 5881 | 14454 ± 5938 | 14509 ± 6357 | 12623 ± 5845 |
| SOM Bias | 6992 ± 7468 | 5016 ± 6241 | 2963 ± 6229 | 698 ± 6552 | -1132 ± 5916 |
| Percent Bias in SOM | 65.11 ± 68.39 | 53.32 ± 64.51 | 41.80 ± 69.77 | 28.43 ± 71.94 | 10.68 ± 68.55 |

* The units of simulation, observation, and bias are in gC·m⁻². The unit of percent bias is %.
